# Evaluation of parents’ awareness about the serious danger related to disc battery ingestion in children

**DOI:** 10.1186/s12887-026-07509-8

**Published:** 2026-08-14

**Authors:** Maria Boccia, Paolo Quitadamo, Rossella Turco, Alessia Salatto, Piergiorgio Gragnaniello, Roberta Buonavolontà, Ileana Bracale, Linda Cosenza, Dorotea Del Buono, Maddalena Chianese, Marco Graziani, Giovanni Di Nardo

**Affiliations:** 1 Department of Translational Medical Sciences, Section of Pediatrics, University of Naples Federico II, Naples, Italy; 2 Pediatric Gastroenterology and Hepatology Unit, Santobono-Pausilipon Children’s Hospital, Naples, Italy; 3 ASL Napoli 1, Naples, Italy; 4 ASL Napoli 2 Nord, Naples, Italy; 5 AUSL Romagna, Forlì, Italy; 6 Department of Neurosciences, Pediatric Unit, Mental Health and Sensory Organs (NESMOS), Sant’Andrea Hospital, Sapienza University, Rome, Italy

**Keywords:** Disc battery, Foreign body, Gastrointestinal

## Abstract

**Objective:**

Disc battery (DB) ingestion is a potentially life-threatening pediatric emergency that requires prompt recognition, timely medical evaluation and urgent management to prevent severe complications. This study aimed to evaluate parents' awareness and knowledge of the risks associated with pediatric DB ingestion.

**Study design:**

A cross-sectional, survey-based study was conducted between March 1 and July 31, 2024, at Santobono-Pausilipon Children's Hospital (Naples, Italy), and at the offices of four primary care pediatricians in Italy. We submitted a 14-item questionnaire to 250 parents of children followed-up for well-child visits and subsequently analysed pooled data derived from their answers.

**Results:**

209/250 (83.6%) parents do not recognize DBs as a dangerous foreign body whenever ingested. Among the different kind of commercially available batteries, only 50/250 (20%) parents consider DBs as the most dangerous. 181/250 (72.4%) parents never heard about the possible risks related to DBs ingestion. Of those who responded that they had heard about the topic, 9/69 (13%) parents claim to have been informed by their pediatrician. In addition, 172/250 (68.8%) parents state that they have never implemented preventive measures at home.

**Conclusions:**

Our data show a very impressive lack of parental knowledge about the risk associated with DB ingestion in children. Many cases of ingestion could probably be prevented if large-scale information campaigns would increase the general population awareness about this source of serious danger to the health and even life of children.

**Clinical trial number:**

Not applicable.

## Introduction

Foreign body (FB) ingestion is a relatively common occurrence in the pediatric population and represents a frequent reason for emergency department evaluation. In children, most episodes are accidental and typically involve objects that are easily accessible in the household environment; it has been estimated that up to 98% of cases occur unintentionally [1–3]. The clinical presentation is highly variable. Some patients develop symptoms such as drooling, dysphagia, chest discomfort, stridor, irritability, abdominal pain, feeding refusal, wheezing, or respiratory distress [4]. In other situations, however, children may remain asymptomatic and are brought for medical assessment because caregivers witnessed or suspected the ingestion.

In the majority of cases, ingested FB progress through the gastrointestinal (GI) tract without causing symptoms or requiring specific treatment. Nonetheless, complications may occur in a minority of patients and can occasionally be severe. Reported adverse outcomes include mucosal ulceration, gastrointestinal bleeding, perforation, mediastinitis, peritonitis, abscess formation, and fistula development. In addition, vomiting may lead to aspiration of the foreign body, with the potential for airway obstruction [5]. From a clinical standpoint, ingested objects are commonly classified into several categories, including blunt objects, sharp or pointed objects, magnets, food boluses, and disc batteries (DBs).

Among these, DB ingestion represents a particularly concerning event. Despite improvements in product safety—such as better-designed battery compartments and warning labels—cases associated with serious injury and death continue to be described [2–8]. DBs are capable of producing rapid tissue damage when they become lodged in the esophagus. Severe complications have been documented, including tracheoesophageal fistula, fistulization involving adjacent mediastinal vessels, massive haemorrhage, and fatal outcomes. Long-term sequelae such as esophageal stenosis, chronic tissue injury, and swallowing impairment have also been reported [9].

The esophagus contains several physiologic narrowings that increase the likelihood of FB impaction, with the thoracic inlet being the most frequently involved location in cases of disc battery retention [10]. Experimental and clinical observations indicate that mucosal injury may begin shortly after impaction, in some cases within approximately 15 min [11, 12]. The resulting tissue damage is mainly related to the generation of hydroxide ions at the battery’s negative pole, which produces a localized alkaline injury due to the rapid rise in pH rather than direct thermal or electrical effects. Because of the potentially catastrophic complications associated with esophageal impaction, current international guidelines recommend prompt endoscopic removal of DBs from the esophagus, followed by appropriate monitoring and management after extraction [13, 14].

Although the clinical risks of DB ingestion are well recognized among healthcare professionals, less is known about how aware parents are of this potentially life-threatening hazard. The present study aimed to comprehensively evaluate parents' awareness, knowledge, preventive practices, and sources of information regarding pediatric DB ingestion, and to investigate their association with parental sociodemographic characteristics, including age and educational level.

## Methods

An observational survey-based study was carried out between March 1 and July 31, 2024, at the Santobono-Pausilipon Children’s Hospital in Naples, a tertiary referral endoscopic Center for the Campania region (Italy), as well as in the offices of four Primary Care Pediatricians in Italy.

A 14-item questionnaire was administered to parents or legal guardians of children aged 0–18 years attending routine well-child visits and scheduled consultations with primary care pediatricians, as well as outpatient appointments at the Pediatric Endocrinology, Clinical Nutrition, Gastroenterology, and Nephrology clinics of Santobono Pediatric Hospital. A total of 250 questionnaires were collected, with one questionnaire completed per family, yielding a final study sample of 250 families. No formal a priori sample size calculation was performed, as this was an exploratory cross-sectional survey aimed at describing parental awareness. A convenience sample was obtained by consecutively recruiting all eligible parents attending the participating outpatient clinics during the study period, resulting in a final sample of 250 participants. No exclusion criteria were applied. The multiple-choice questionnaire was designed to assess parents’ knowledge of DBs, their awareness of the potential health risks associated with DB ingestion in children, whether they had previously received information or warnings on this topic and from which sources, and whether they had implemented any preventive measures within the home environment.

The data obtained from the questionnaires were subsequently collected and analyzed. Study data were recorded in Excel spreadsheets (Microsoft Inc., Washington, USA) and analyzed using GraphPad PRISM software version 5.1 (GraphPad Software Inc., California, USA) and the R statistical computing environment version 3.6.0. Continuous variables were expressed as mean ± standard deviation, whereas categorical variables were reported as frequencies and percentages. Statistical comparisons were performed using the χ^2^ test or Fisher’s exact test, as appropriate, to evaluate differences in clinical presentation (symptomatic vs. asymptomatic). A *p*-value ≤ 0.05 was considered statistically significant, and odds ratios were reported with 95% confidence intervals.

The study was approved by the local ethics committee “Comitato Etico Campania 1” (reference number 2024/461–2) and was conducted in accordance with the Declaration of Helsinki [15] and Guidelines for Good Clinical Practice [16]. In full compliance with the current privacy regulation, personal patient demographic data were not recorded. Informed consent to participate was obtained from the parents or legal guardians of any participant under the age of 16.

## Results

Over the study period 250 questionnaires were collected. The majority of responders were 31–40 year-old parents (126/250, 50.4%), whereas 70/250 (28%) were 20–30 year-old, 41/250 (16.4%) were 41–50 year-old, 12/250 (4.8%) were > 50 year-old and 1/250 (0.4%) was < 20 year-old. Most responders (113/250, 45.2%) had a high school diploma, 79/250 (31.6%) had an eighth-grade education, 54/250 (21.6%) were graduated and 4/250 (1.6%) had an elementary school diploma.

Most parents (177/250, 70.8%) claimed to know about the existence of DBs and, when displayed, 231/250 (92.4%) of responders could correctly identify them. However, 181/250 (72.4%) parents have never heard about the possible health risks related to DB ingestion. Indeed, among several FBs (coin, nail, disc battery, piece of glass, pen drive), 209/250 (83.6%) parents did not consider DBs the most potentially dangerous FBs, whenever ingested. Moreover, even among the different kinds of commercially available batteries, only 50/250 (20%) parents consider DBs as the most dangerous (Table 1).

**Table 1:** Parents’ answers on their knowledge about DB and their awareness about possible danger related to their ingestion

**Do you know about the existence of disk batteries?**	**n (%)**
Yes, I do	177 (70.8%)
No, I don’t	34 (13.6%)
I’m not sure	39 (15.6%)
**Have you ever heard about the possible risks related to disk battery ingestion?**	
Yes, I have	69 (27.6%)
No, I have not	181 (72.4%)
**Which battery do you think is more dangerous if swallowed?**	
Disk batteries	50 (20%)
Cylindrical batteries	64 (25.6%)
I think disk batteries and cylindrical batteries are equally dangerous	136 (54.4%)
**Do you know which of the devices in your home environment contain disk batteries?** **(toys, household utensils, electronic devices etc.)**	
Yes, I do	128 (51.2%)
Yes, quite	62 (24.8%)
No, I don’t	32 (12.8%)
Maybe	28 (11.2%)

When asked about what would they do if their child had ingested a DB, 159/250 (63.6%) of them would immediately take him to the emergency room, even though he had no symptoms; 57/250 (22.8%) of parents would only take him to the emergency room if developing symptoms; 10/250 (4%) of them would observe him for a few hours and eventually alert the family pediatricians; 19/250 (7.6%) of them would try to induce vomiting; finally, 5/250 (2%) would only check the stools over a few days to look for the battery.

As a consequence of their poor awareness, 172/250 (68.8%) parents stated that they had never implemented preventive measures at home in order to avoid DB ingestions by their children. Moreover, only about half of them (51.2%) consider themselves aware of which devices in their home environment may actually contain DBs (toys, tools, electronic devices etc.).

Among parents who were aware of the potential risks associated with DB ingestion, only 9 of 69 (13%) reported having received information from their primary care pediatrician. The remaining parents instead reported obtaining information from non-medical sources, including their child's school, the internet, television, social media, and friends or relatives.

According to 242/250 (96.8%) parents, it would be necessary to provide parents with more extensive and detailed information about this serious health hazard.

**Fig. 1:**
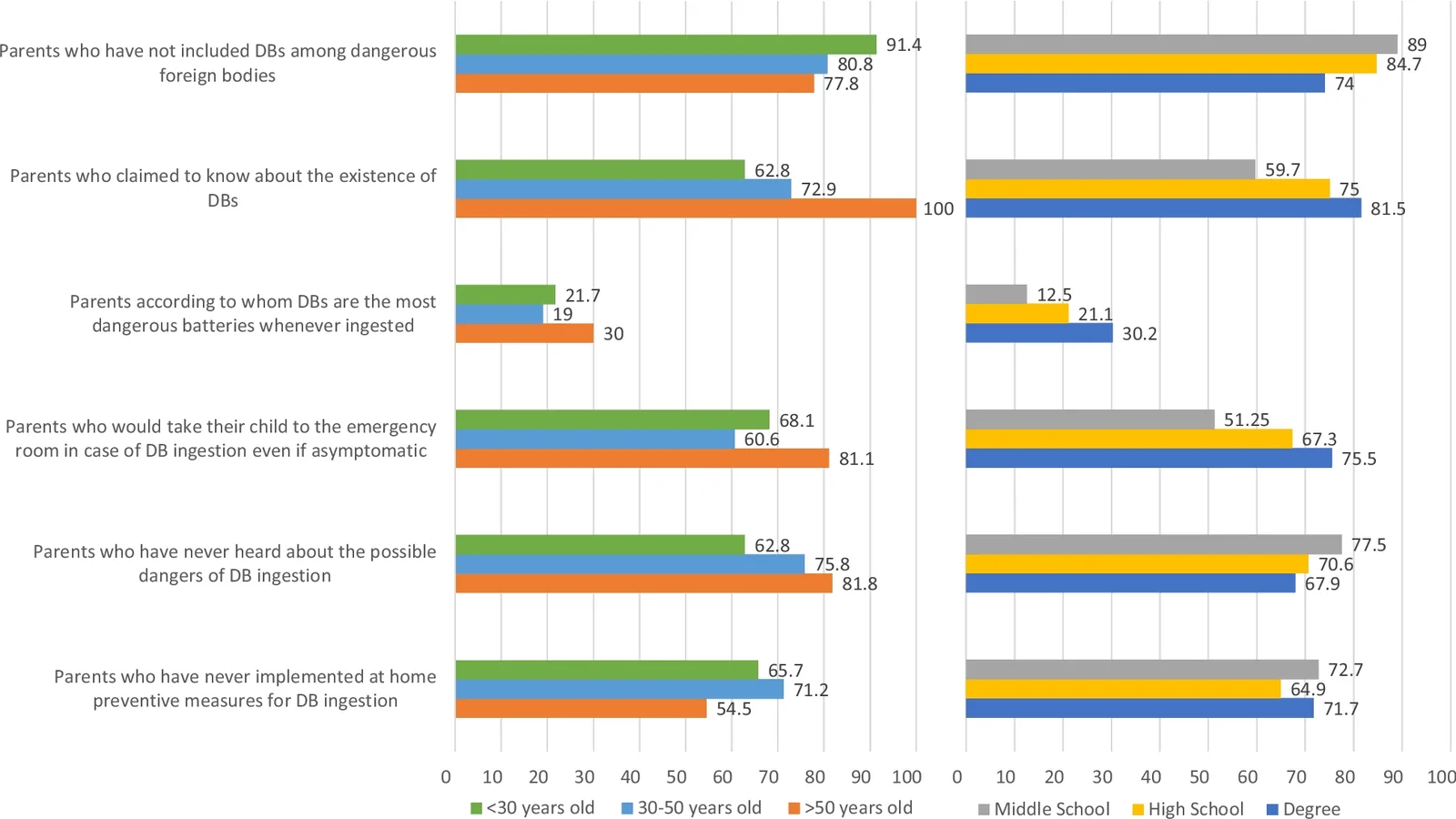
Parents’ knowledge about DBs stratified by age and education. Results are expressed in percentiles

Among the parent’s subgroups with different age and level of education, graduated parents and parents aged > 50 years provided the correct answers in higher percentages. Of note, graduated parents were significantly more aware than parents with a lower education level about DB existence and health threat by ingested DB and showed a better knowledge of the correct management of children having ingested DBs. Finally, the only statistically significant difference among the answers of parents from different age groups was reported for the item exploring DB existence knowledge (Fig. 1). The data is sometimes contradictory. For example, the highest percentile of parents who claim to know about existence of DBs is the > 50 years population, but that same population also comprises the majority of parents who have never heard about the possible dangers of DB ingestion.

## Discussion

Despite the growing incidence and the possible dramatic consequences of DB ingestion by children, along with the critical role of parental vigilance in mitigating this risk, only a few studies have explored parental awareness of this issue. These studies have been carried out among Australian and North American populations. Therefore, to date data related to European caregivers are still missing.

Our study helps address this knowledge gap by providing detailed insights into a sample of Italian caregivers, which may inform the development of targeted educational interventions and safety recommendations in the field of foreign body ingestion, particularly the prevention and management of DB ingestion.

Our data, collected from a large sample of specifically questioned parents, reveal a noteworthy lack of knowledge about the risks associated with DB ingestion in children, suggesting that the general population is currently inadequately informed, despite awareness has increased within the medical community.

One of the main findings of our survey, which investigated several aspects of the issue, is that most parents are unaware of the health hazards linked to DB ingestion and have never received adequate information. Of note, almost three out of four caregivers reported having never heard about these possible risks. Therefore, only a small proportion of parents recognize DBs as the most hazardous type of ingested FB or at least as the most dangerous among commercially available batteries.

A similar but even more impressive result concerns how would parents act in case of DB ingestion by one of their children. Although the majority of them stated they would take their child to the emergency department immediately following DB ingestion, regardless of symptom development, about one out of four would have some hesitation in requiring immediate medical advice, thus severely reducing the chances of a favourable clinical outcome.

As a consequence of this lack of awareness, 2 out of 3 parents reported they had never implemented preventive measures at home to reduce the risk of DB ingestion. A near-unanimous consensus emerged among parents about the need for more comprehensive and detailed information of the serious health hazards posed by DB ingestion.

Our findings do not align with prior research studies, which reported much higher levels of awareness and knowledge by parents. In 2024 *Girardi *et al. published the results of a cross-sectional online survey disseminated through social media in Australia [17]. Their sample included caregivers of children aged 0–5 years and was recruited via convenience sampling. Up to 98% of the surveyed caregivers acknowledged the potentially fatal outcome of DB ingestion. These positive findings are likely a consequence of media coverage of severe cases and effective Australian awareness campaigns, which are likely still insufficient in our regions.

Over the same years, *Philteos *et al. carried out a similar cross-sectional online survey with 930 participants recruited via social media, mostly from North America [18]. Quite in line with *Girardi *et al.'s findings, the majority of participants (80%) recognized that DBs can cause severe injury and 61% acknowledged their potential lethality, as well. Moreover, compared to our study, a larger proportion of respondents would seek immediate emergency care after DB ingestion, regardless of symptom development. These results demonstrate a greater level of awareness and knowledge of the issue compared to those observed in our study. However, *Philteos *et al. reported comparable findings about the level of preventive practices adopted by the responders, reinforcing the need to improve public understanding of prevention.

A notable point of agreement between the two studies is the widespread call for improved education. In our sample, 96.8% of respondents expressed the need for more detailed information. Likewise, *Girardi *et al. sample stressed the value of sustained public education campaigns to address knowledge gaps and promote safer parental behaviours and *Philteos *et al. observed strong demand for better education, as well.

Furthermore, a more detailed statistical analysis by subgroups allowed us to highlight how parental knowledge appeared significantly influenced by both education and age. Specifically, parents over the age of 50 and those with a university degree provided more accurate responses compared to other subgroups. Indeed, our analysis found that parental age and education positively correlates with awareness. Similarly, *Philteos *et al. noted that socioeconomic status influenced DB knowledge: only 50% of respondents with annual incomes under $10,000 were aware of the potential fatality of DB ingestion, compared to 70% among those with higher incomes.

We acknowledge that our study has some methodological limitations. First of all, the study was carried out across the outpatient clinics of four primary care pediatricians located in different Italian regions, yet without the claim of homogeneously representing the entire Italian population. As a consequence, this may restrict the generalizability of our findings. Additionally, we have not administered a validated questionnaire, as no such tool is currently available, and recruitment was based on convenience sampling within clinical settings, which may have introduced a selection bias. As a result, the study sample lacked a balanced distribution across age and education strata, with a predominance of respondents aged 31–40 years and those holding a high school diploma. This may have limited the representativeness of subgroup analyses based on age and educational level. Furthermore, we did not collect information on the age of participants' children at the time of questionnaire administration. Consequently, we were unable to determine whether parental awareness varied according to the child's age, particularly among families of children younger than 5 years, who are at the highest risk of disc battery ingestion. Future studies should incorporate child age to identify potential knowledge gaps across age groups and to better inform the development of targeted educational interventions for families of children at greatest risk.

In conclusion, DB ingestion represents a significant health hazard that should not be underestimated. Rather, the risk and severity of injuries caused by DBs warrant ongoing research into the prevention of such events and their medical management [19]. According to our data, parental awareness about the associated risks, appropriate post-ingestion management and preventive strategies are currently inadequate in our regions. Large-scale educational initiatives, particularly those involving primary care pediatricians, may play a crucial role in reducing the incidence of these potentially life-threatening events.

However, education alone is unlikely to be sufficient. Effective prevention also requires limiting children's access to DB through safer product design, secure battery compartments, child-resistant packaging, and appropriate regulatory measures. Companies manufacturing DBS should be mandated to put warning signs on their products and adverts. In this regard, legislative initiatives such as Reese's Law in the United States have introduced mandatory safety standards for consumer products containing button or coin batteries, representing an important complementary strategy to public education (https://www.reesespurpose.org/reeseslaw). Similar regulatory approaches, alongside caregiver education, may further reduce the burden of disc battery ingestion.

Increasing such efforts could enhance public understanding of this serious risk and contribute to safeguarding the health and lives of children.

Despite its limitations, the value of our study is to address the current paucity of parental awareness of the serious danger associated with DB ingestion in children and to provide data from a geographic area where published research is very limited, bringing to light issues that deserve further investigation. Although efforts to reduce DB hazards in the home environment must also address production and design practices, this study may raise awareness of the parent and caregiver role in DB safety.

## Data Availability

Availability of data and materials: Yes, upon request. Contact details: Paolo Quitadamo, MD, Pediatric Gastroenterology and Hepatology Unit Santobono-Pausilipon Children’s Hospital, Via Mario Fiore 6, 80,129—Naples, Italy, e-mail: paoloquitadamo@yahoo.it.

## Abbreviations

DB: Disc Battery
FB: Foreign Body
GI: Gastrointestinal
